# The role of calcium oscillations in the phenotype selection in endothelial cells

**DOI:** 10.1038/s41598-021-02720-2

**Published:** 2021-12-10

**Authors:** Birses Debir, Cameron Meaney, Mohammad Kohandel, M. Burcin Unlu

**Affiliations:** 1 Department of Physics, Bogazici University, 34342 Istanbul, Turkey; 2 Department of Applied Mathematics, University of Waterloo, Waterloo, ON Canada; 3 Hokkaido University, Global Station for Quantum Medical Science and Engineering, Global Institution for Collaborative Research and Education (GI-CoRE), Sapporo, 060-8648 Japan

**Keywords:** Computational models, Computational biophysics

## Abstract

Angiogenesis is an important process in the formation and maintenance of tissues which is driven by a complex system of intracellular and intercellular signaling mechanisms. Endothelial cells taking part in early angiogenesis must select their phenotype as either a tip cells (leading, migratory) or a stalk cells (following). Recent experiments have demonstrated that rapid calcium oscillations within active cells characterize this phenotype selection process and that these oscillations play a necessary role in governing phenotype selection and eventual vessel architecture. In this work, we develop a mathematical model capable of describing these oscillations and their role in phenotype selection then use it to improve our understanding of the biological mechanisms at play. We developed a model based on two previously published and experimentally validated mathematical models of calcium and angiogenesis then use our resulting model to simulate various multi-cell scenarios. We are able to capture essential calcium oscillation dynamics and intercellular communication between neighboring cells. The results of our model show that although the late DLL4 (a transmembrane protein that activates Notch pathway) levels of a cell are connected with its initial IP3 (Inositol 1,4,5-trisphosphate) level, cell-to-cell communication determines its eventual phenotype.

## Introduction

Angiogenesis is the process by which existing vasculature sprouts new vessels to alleviate areas of oxygen deficiency. It is essential for the development, maintenance, and survival of tissues and plays an essential role in many disorders. In particular, the mechanisms governing angiogenesis are frequently dysfunctional in cancerous tissues, leading to improper cell-to-cell angiogenic signaling and an abnormal vascular network. Deregulated angiogenic signaling is accepted as one of the fundamental hallmarks of cancer^1,2^, and many modern anticancer treatments or studies are designed to target aspects of this dysfunctional vasculature^3–6^. However, resistance to these anti-vascular therapeutics or activation of compensatory pathways is common, creating a need for anti-angiogenic therapies which rely on novel substances^7,8^. For example, calcium-regulating treatments have recently been explored as potential anti-angiogenic therapies^7^. As a second messenger, calcium has a multifunctional role in cell communication. For cancer specifically, calcium plays a crucial role in relevant pathways involved in angiogenesis, metastasis, and local cancer progression^9–12^. Recent experiments have more thoroughly investigated the link between cytosolic calcium oscillations in endothelial cells (ECs) and the proangiogenic molecule vascular endothelial growth factor (VEGF)^13,14^, revealing the importance of Ca2+ oscillations to cell differentiation in the early stages of angiogenesis. Since positive regulators of angiogenesis are still under investigation^15^, calcium should also be investigated as a spatio-temporal signalling modulator.

Growth factors, such as VEGF, secreted from cells in low oxygen environments act as signals which cause quiescent, unpatterned (ECs) to activate and begin vessel formation. However, for each sprouting vessel, the participating ECs must undergo a competition process to decide which will lead the new vessel and which will follow along. Calcium oscillations are present in this cellular competition and are key players in determining which cell will begin to bud from the existing vessel. The angiogenesis signaling pathway is complex and interconnected with other cellular signaling pathways. Here, we only focus on the parts of the pathway essential for understanding our mathematical model and its application. VEGF signaling triggered by oxygen-deficient cells causes an upregulation of intracellular $\text {PLC}\gamma$, which in turn increases levels of inositol 1,4,5-trisphosphate (IP3). IP3 upregulation causes the IP3 receptors (IP3R) present on the surface of the endoplasmic reticulum to open, leading to a rapid increase in cytosolic Ca2+. Cellular homeostasis of calcium ions is maintained through various exchangers and pumps as part of the extracellular signal-regulated kinase (ERK) pathway, which causes the secretion of delta-like ligand 4 (DLL4) as a byproduct. DLL4 is a transmembrane protein that interacts with the Notch protein on the surface of neighboring cells. If DLL4 of a neighbouring cell dimerizes Notch, it releases Notch intracellular domain (NICD) into the cytoplasm, which inhibits the VEGF pathway through the Hairy and Enhancer-of-split (HE) family of proteins. Here, Notch target factors (Hes, Her, Hey) are gathered as HE family for expressing all resulting from the VEGFR cascade repressors together^16–18^. This process of cells activating their VEGF pathways while simultaneously inhibiting neighbouring pathways gives rise to the previously mentioned competition mechanism where a few cells emerge from the many, leading to the formation of the new vessel branch.

As the cell-to-cell competition progresses, ECs become distinguished based on the activation of VEGF downstream factors. Cells with inactive downstream factors will attach and follow cells with active factors, proliferating and migrating away from the vessel. This characterization brings about two distinct phenotypes: the ’tip’ phenotype - proliferative and migratory cells at the tip of the new branch - and the ’stalk’ phenotype - proliferating but follower cells at the base of the new vessel branch. In addition to the behavioral differences between tip and stalk cells, tip cells can also be distinguished by their prominent and dynamic filopodia^19^. These long thin actin protrusions exist in higher quantities in tip cells, increasing their migratory abilities and ability to sense the direction of enhanced signaling^20^. Furthermore, tip and stalk cells are patterned such that neighboring cells cannot both display the tip phenotype. Under extracellular VEGF, all cells initially exhibit a tip phenotype but later readjust their phenotype following an inhibitory process^21^.

The EC phenotypic selection process is achieved through lateral inhibition of the VEGF signaling pathway between neighboring ECs on the mother vessel through DLL4-Notch interaction on cell surfaces. In theory, cells with a high level of DLL4 tend toward the expression of a tip phenotype with increased mobility features such as filopodia, and cells with a lower level of DLL4 tend to express the stalk phenotype^22–24^. In reality though, the temporal dynamics of this phenotypic separation are not this simplistic and can be linked to other initiators as well, such as oscillating intracellular signals.

Temporal analysis is crucial in complex processes, and this is especially true in the context of angiogenesis which includes multiple processes such as cell migration, proliferation, and phenotype selection. Recent works have inspected the temporal scales of angiogenic decision-making in this context. Venkatraman et al.^25^ designed a two-cell model coupled through VEGF-Notch-DLL4 system where they investigate the variations of time spent in phenotype identities. They examined how the temporal decisions on cellular identities are affected by local conditions. They built a two-cell model having positive feedback with rate equations for intracellular VEGF, VEGFR, DLL4, Notch, DLL4- Notch complex, NICD, HE, and filopodia. By using time-lapse imaging of zebrafish embryos, Yokota et al.^13^ showed VEGF-dependent Ca2+ oscillations during endothelial phenotype selection. They observed that all VEGF-activated ECs contained oscillations in their concentration of Ca2+, but that these oscillations survived only in the budding cells, which took on the tip cell phenotype. Savage et al.^14^ proposed that Transmembrane protein 33 (Tmem33) was required for cytosolic calcium oscillation in activated ECs. They showed that Tmem33 knockdown reduced ERK phosphorylation and Notch expression in zebrafish and human ECs. They also observed that calcium oscillations maintain filopodia extension and migration. Also, Ca2+ signaling is modulated in mammalian cells with dose-dependent VEGF signaling network^26^. Various cellular responses are thought to be conveyed by temporal Ca2+ dynamics; however, the precise outcome of angiogenic calcium signaling on endothelial populations requires further investigation.

The versatility of angiogenesis is built upon a delicate balance, usually depicted via the DLL4/Notch pathway. Absent from this model however is the notion of migrating cells moving along the gradient of external signals. To incorporate cell migration into a model, one must necessarily consider the effects of calcium and its multi-faceted role in migration. This work aims to model the relationship between tip cell selection and overall calcium transients in the cell. Although Ca2+ has a complex function in cell migration, our model meets the fundamental features of phenotype difference without referring to calcium in cell migration. To our knowledge, this is the first analytical model for investigating the role of calcium in phenotype characterization for angiogenesis. Here, we initiated a calcium-related lateral inhibition process in unpatterned ECs. We considered implicit VEGF intake by cells dictated by IP3 concentrations and explored the relationship between VEGF-related calcium oscillations and DLL4 levels. Our model results agree with experimental findings exploring a correlation between phenotype identity and calcium oscillations since cells containing high levels of VEGF sustained Ca2+ oscillations are categorized as having the tip phenotype. We further explored this idea in multiple-cell scenarios for comparing how ECs are patterned by analyzing how cell-to-cell interaction between the neighbors influenced both the calcium levels and phenotype selection outcome.

## Methods

### Model

Temporal regulation of the DLL4/Notch pathway modulates the vascular architecture^25,27,28^. In their experimentally validated early angiogenesis model, Venkatraman et al.^25^ investigated tip cell competition by VEGFR-mediated DLL4/Notch interaction. In their model, two adjacent cells compete against each other by negative lateral inhibition feedback and positive feedback with an arbitrary feedback parameter. Originating from the observations of Yokota et al.^13^, we exclude the early factors of DLL4 and connect DLL4 concentration directly to cytosolic calcium.

Selection of the tip cell phenotype activates migratory features within a cell. In addition to the usage of Ca2+ in cellular communication, Ca2+ is also involved in multiple other parts of cell migration. To avoid unnecessary complexity, our model contains the model in Atri et al.^29^, representing calcium dynamics only via the IP3R and Sarco/endoplasmic reticulum Ca2+-ATPase (SERCA) dynamics on the Endoplasmic Reticulum (ER). The model takes advantage of the calcium induced-calcium release (CICR), which creates Ca2+ transients when the model includes a diffusion term for Ca2+. This non-excitable cell model is used for displaying calcium dynamics in different cell types for a wide range of problems^30–36^. Wide usage of the model is based on its effectiveness and simple logic. IP3 receptors interact with dynamic cytosolic calcium and IP3, μ, which is adapted as a parameter for gauging oscillations. For a range of μ values, oscillatory behavior in Ca2+ concentration can be generated in the system. The rich wave dynamics produced by the model are enabled by the different timescales between activation and inactivation of receptor dynamics.

**Figure 1:**
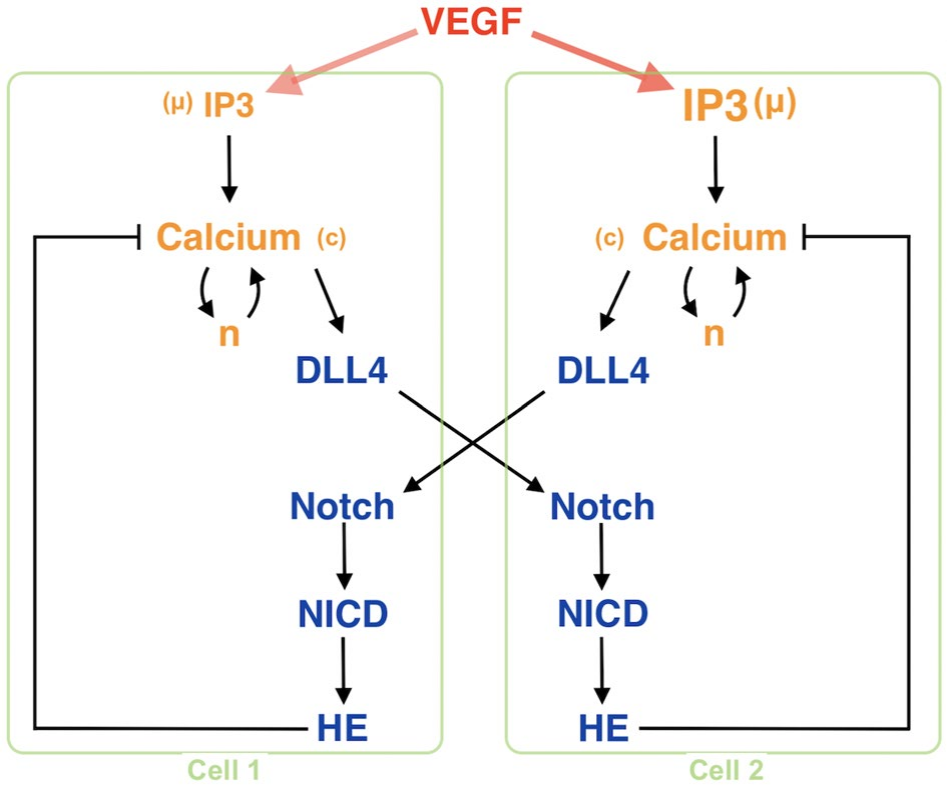
Schematic representation of the two cell model explaining the relevant factors and the interactions between them. Here, each cell is illustrated as green boxes including VEGF downstream pathways. VEGF resides in the extracellular space and is included through cellular IP3 concentration through $\text {PLC}\gamma$. In this model, we are aiming to compare the effect of varying VEGF intakes on a cell’s angiogenesis dynamics. For this reason, we keep every variable and the initial conditions the same except for VEGF levels. Since IP3 levels are associated with VEGF over $\text {PLC}\gamma$, the only difference between the cells is accepted as being IP3 levels. Calcium related factors (yellow) are cytosolic calcium, *c*, the proportion of IP3 receptors not inactivated by calcium, *n*, and IP3 concentration, μ. The Ca2+ model includes the interplay between IP3R and SERCA pump. Additionally, angiogenesis-related factors (blue) are DLL4, Notch, NICD, and HE. Being transmembrane proteins, DLL4 and Notch interact with each other to generate a DLL-Notch complex which is also included in the model. Furthermore, while HE family factors mediate Ca2+ by negative feedback, DLL4 secretion is connected to calcium concentration.

The cellular pathway presented in Fig. 1 is a cytoplasmic calcium-based tip cell model that defines tip cell phenotype as high DLL4 concentration. Cells are represented spatially by a one-compartment model and a temporal model. All variables (Table 1) and coefficients in the below are in dimensionless units.

**Table 1:** The dimensionless parameters which were taken from^25,29,31^.

Parameter	Symbol	Value	References
Diffusivity of Ca2+	$D_{0}$	0.1	^29^
Basal gene expression of proteins	$\beta$	0.001	^25^
Production rate of NICD	$\theta$	0.1	^25^
Production rate of DLL4	$\Theta$	1.5	Estimated
Negative feedback rate of HE on Calcium	$\zeta$	0.6	Estimated
Basal current through IP3R	*b*	0.111	^29^
Maximum total Ca2+ flux through IP3R	$k_{1}$	23.1	^31^
Maximmum rate of Ca2+ pumping from the cytosol	$k_{2}$	5.7	^29^
Half maximum rate of Ca2+ pumping from the cytosol	$k_{3}$	0.14	^29^
Half maximum rate of DLL4 production	$k_{4}$	1.4	^25^
Association rate for dn complex	$k_{5}$	0.4	^25^
Disassociation rate for dn complex	$k_{6}$	0.002	^25^
Degradation rate of proteins	$k_{7}$	0.01	^25^
Catalyses rate for dn complex	$k_{8}$	0.2	^25^
Half maximum rate of NICD production	$k_{9}$	0.5	^25^

They are either phenomenological or estimated by experiments. $\Theta$ and $\zeta$ values are estimated in this model to represent the observations of Yokota et al.^13^.

In the two-cell model equations depicted below, the subscript *i* specifies the cell while the subscript *j* specifies its neighbour. Calcium dynamics are governed by the following equations,

1 $$\begin{aligned} \frac{ {\partial } c_{i} }{ {\partial } t}&= {D_{0}}\;{\nabla ^2} c_{i} + {k_{1}}\;\mu _{i}\; n_{i} \left( \frac{b + c_{i}}{1 + c_{i}}\right) - {k_{2}}\; \frac{c_{i}}{{k_{3}} + c_{i}} -\zeta \; z_{i}\; c_{i} \end{aligned}$$

2 $$\begin{aligned} \frac{ d n_{i} }{d t}&= 1 - \frac{c^2_{i}}{1 + c^2_ {i}} - n_{i} \end{aligned}$$

where *c* and *n* correspond to the cytosolic calcium concentration and the proportion of IP3 receptors not inactivated by calcium. The terms on the right-hand side of the calcium equation represent calcium diffusion inside the cytosol, endoplasmic Ca2+ flow by IP3 receptor, cytoplasmic calcium pumped out, and negative feedback by HE family proteins on calcium. Here, we examine a situation where calcium dynamics are activated only due to a VEGF-related increase in cytosolic IP3 levels. Concomitantly, Ca2+ levels diminish by efflux from the cytosol and HE negative feedback. Repression to the signaling cascade is applied on Ca2+ rather than IP3 since inhibited signals are observed in Yokota et al.^13^. In addition, the right-hand side of Eq. (2) denotes receptor inactivation by Ca and receptors.

Stable configurations in EC phenotypes are usually distinguished by a specific gene expression of DLL4^25,37^. Here, we follow the same logic and consider DLL4 concentration as the sole determiner of expression of the tip cell phenotype. The equations describing the interaction between adjacent cells are as follows,

3 $$\begin{aligned} \frac{ d v_{i} }{d t}&= \beta + \Theta \;\frac{c^2_{i}}{{k_{4}}^2 + c^2_{i}} - {k_{5}}\; v_{i} \; w_{j} + {k_{6}}\;x_{j} - {k_{7}}\; v_{i} \end{aligned}$$

4 $$\begin{aligned} \frac{ d w_{i} }{d t}&= - {k_{5}}\; v_{j} \; w_{i} + {k_{6}}\; x_{i} - {k_{7}} \;w_{i} \end{aligned}$$

5 $$\begin{aligned} \frac{ d x_{i} }{d t}&= {k_{5}}\; v_{j} \; w_{i} - {k_{6}}\; x_{i} - {k_{7}}\;x_{i} \end{aligned}$$

where, *v*, *w*, and *x* describe concentrations of DLL4, Notch, and DLL-Notch complex, respectively. The terms in Eq. 3 are the basal DLL4 expression, the gene expression of DLL4 by Ca2+, DLL4 association by Notch of the adjacent cell, disassociation of the complex of the neighbour and DLL4 degradation. Respective terms in Eq. 4 are an association of adjacent cell’s DLL4 with Notch, disassociation of the DLL-Notch complex and Notch degradation. In addition, the terms on the right-hand side of the complex equation are an association of adjacent cell’s DLL4 with Notch, disassociation of the complex and degradation of the complex.

Following DLL4-Notch signalling between neighbouring cells, the equations depicting NICD triggered secretion of HE family proteins are given by

6 $$\begin{aligned} \frac{ d y_{i} }{d t}&= {k_{8}}\; x_{i} - {k_{7}}\;y_{i} \end{aligned}$$

7 $$\begin{aligned} \frac{ d z_{i} }{d t}&= \beta + \theta \;\frac{y^2_{i} }{y^2_{i} + {k_{9}}^2 } - {k_{7}}\;z_{i} \end{aligned}$$

where *y* and *z* describe NICD and HE concentrations in the cell. The terms on the right-hand side of Eq. 6 are the catalysis of the complex, and degradation of NICD. The terms in the last equation are the basal secretion of the factor, gene expression of HE by NICD, and HE degradation.

Initial conditions used in the model were taken from^25,29^. The initial values of *c*, and *n* ($c_0 = 0.200165$ and $n_0=0.961477$) provide the special conditions for the spatially observed wave dynamics in calcium transients. In addition, *v* value ($v_0 = 0.1$) indicates DLL4 concentration preexist any external trigger of the DLL4 - Notch pathway. At last, the rest of the variables have no initial concentrations for this model.

### Numerical simulations

A square domain with side length $x=30$ for time $t= 2000$ was considered for each cell when solving our model numerically. Characteristic variables for our model are $x=x_0 \tilde{x}$ and $t=t_0 \tilde{t}$ for nondimensionalization where *x*_0_ = 20 μm and $t_0=2$. The spatial two-cell model is solved using a finite element method implemented using the FEniCS Project Software^38,39^. A Crank-Nicolson time-stepping scheme is used to discretize the temporal domain for all equations. In the experiment by Yokota et al.^13^, both Ca2+ transients are observed across the cell, and cytosolic calcium changes are measured from the fluorescence of a calcium-related dye for a specific region of individual cells. Therefore, the experiment yields two types of information: spatial Ca2+ transients and temporal oscillations. Here, we aimed to reproduce both the spatial and temporal measurements. Considering that DLL4 and Notch factors are transmembrane proteins that should reside on the boundary of the surface, the setup of Venkatraman et al.^25^ isn’t suitable for a spatial domain simulation. Therefore, we used a spatial and temporal model. All initial conditions for PDE Ca2+ equation are taken the same as in the temporal case. Ca2+ is given an initial condition of a localized Gaussian peak at the center of the cell, capable of initiating the traveling wave dynamics.

For the temporal two-cell setup, the diffusion term in 1 is excluded, such that the equation governing the Ca2+ dynamics becomes an ODE. The model simulations are performed using Method of Lines with a built-in adaptive solver NDSolve, in Mathematica 12. This two-cell setup clearly shows the effect of DLL4 - Notch coupling. Without coupling, we do not have the resurrecting secondary oscillations resulting in the difference in phenotypes.

In the multi-cell setup, the vessel is accepted as it consists of a total of six cells in a 2 × 3 setting. We perform simulations for all cells simultaneously. Since each cell has seven rate equations, we have 42 equations coupled through DLL4 - Notch signaling with different IP3 levels in a multi-cell scenario. Even though this model focuses only on temporal values, we placed cells such that an azimuthal cross-section of the vessel comprised two cells. The alignment and the enumeration of the cells are given in Fig. 4. Similar to the spatial calcium transient domain, cells in the multi-cell model are accepted as squares, and their placement is shifted. This arrangement enables equal contact sites with a different number of neighbors. This multi-cell setup depicts the result of cell-to-cell communication in vascular patterning. Patterning in neighboring ECs is examined under different conditions, which cannot be created with a two-cell setup only. We use six cells for examining the effects in a small region of cells. We focus on this close neighborhood for investigating the effects of cell-to-cell contact on a cell rather than examining the collective behavior which can be made with multicellular models including a higher number of cells. With this 6-cell set up, the model behaviour under several scenarios are examined. The first of these scenarios includes an IP3 gradient. The second focuses on the results of an individual increase in IP3 levels of a cell, while keeping its neighbors at the same concentrations. The last investigates a scenario where two high IP3 cells with the same concentration are neighbours.

## Results

Using in silico models in this paper, we attempt to reproduce the results observed in the experiments detailed above. In particular, we aim to create a model capable of describing calcium oscillations coupled with angiogenic processes based on a lateral inhibition mechanism involving DLL4/Notch interaction. Since inhibition is contact-dependent, we propose several set-ups for cell domains: (i) a two-cell spatial configuration for inspection of Ca2+ distribution, (ii) a two-cell model with temporal dynamics, and (iii) a 6-cell set-up for investigation of neighboring dynamics. We also consider the interaction of the lateral inhibition mechanism with HE-calcium negative feedback to inhibit oscillatory dynamics in the cell. Studies investigating angiogenic Ca2+ oscillations^13,26^, and VEGF signalling network^25^ use procedurally-defined arbitrary units. Here, the model is also combined and presented using dimensionless units. So far, experiments looking at early angiogenesis have not reported cellular concentrations of relevant molecules. Therefore, in their absence, cell phenotypes can be assumed based on other traits: for example, in the experiment by Yokota et al.^13^, tip cells are defined according to their shape rather than their DLL4 concentration.

### Calcium Transients

**Figure 2:**
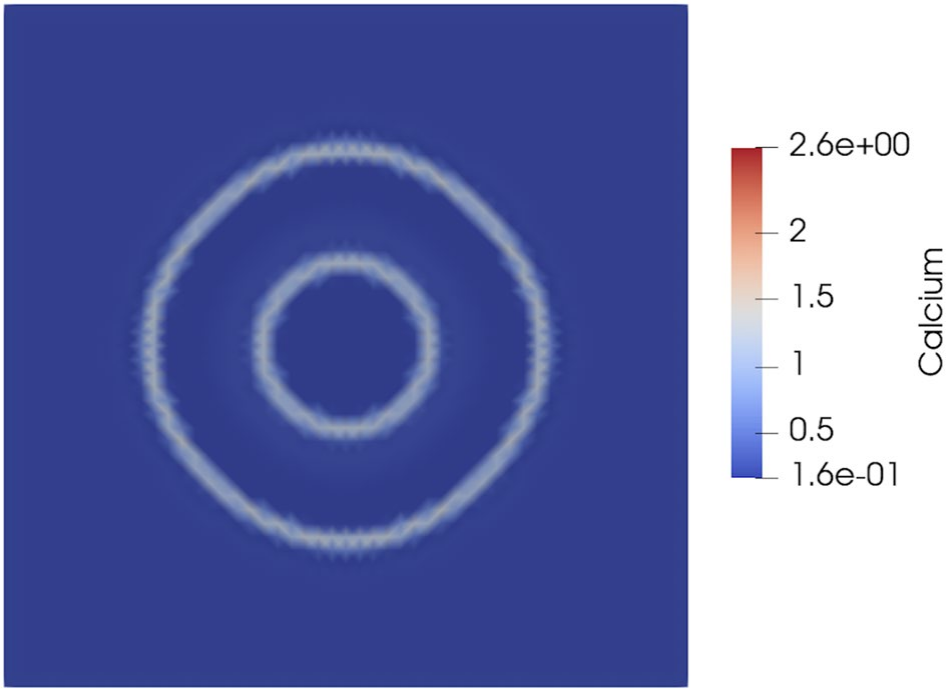
Spatial configuration for Ca2+ dynamics in the presence of diffusing from the Gaussian peak at the center. Configuration is estimated for $\mu = 0.28900$ in 2. At $\mu = 0.28900$, a Hopf bifurcation occurs, and relaxation oscillations are observed. Reaction of system to low IP3 levels and corresponding Ca2+ wave dynamics.

To evaluate whether our model can present Ca2+ transients as observed in vivo in the experiments by Yokota et al^13^, we performed simulations in a spatial domain with calcium diffusion. We focused on how wave dynamics change with IP3 and VEGF intake considering calcium transients influence filopodia and other migrational features of the cell. The model is capable of creating calcium transients on a spatial domain. We evaluated the model with Ca2+ diffusion and a localized Gaussian peak at the center of the domain to initiate the wave dynamics. The transients that our model predicts are consistent with observations. Notably, we also observed that before the calcium waves were suppressed by negative HE feedback, traveling waves were observed in cells with low levels of IP3. For cells with higher levels of IP3, the traveling waveform loses its interesting traveling features and becomes a step-like wave. Fig. 2 depicts how Ca2+ waves propagate from the center of the cell toward its boundaries. When VEGF intake and therefore cytoplasmic IP3 concentration is high, the balance between diffusion and reaction dynamics is distorted on behalf of reaction, and the interesting features of the wavefront are lost.

### Two-Cell Temporal Model

**Figure 3:**
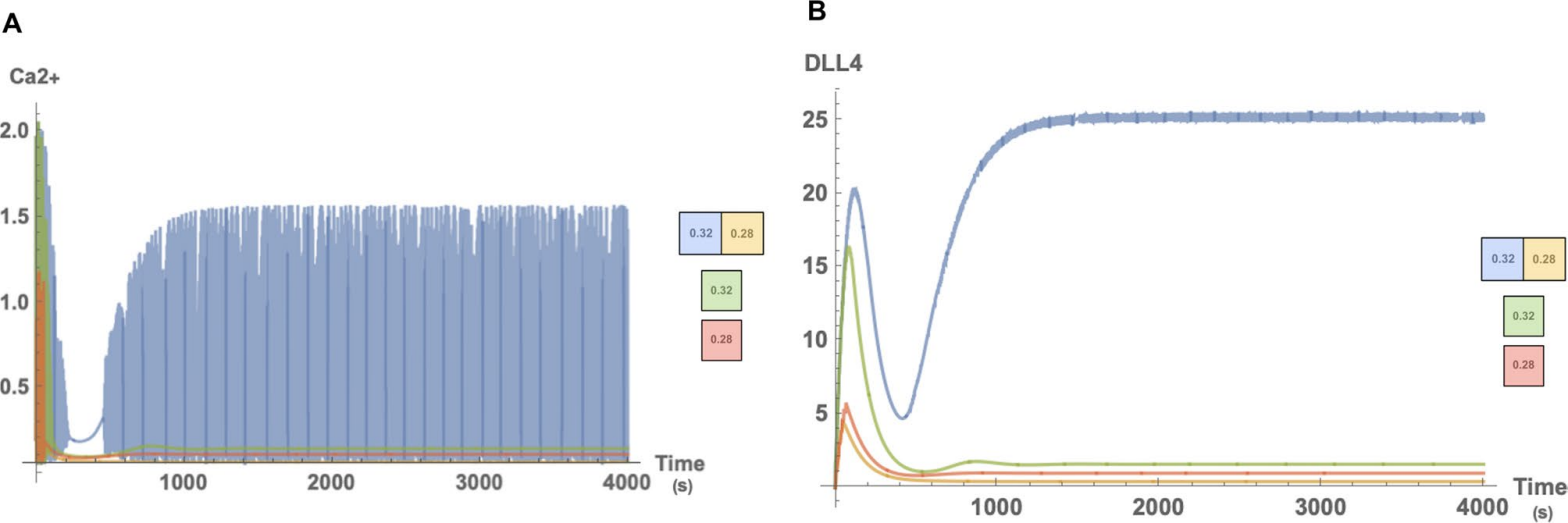
For $\mu _{left}=0.29$ and $\mu _{right}=0.32$ temporal calcium and DLL4 dynamics. In (**A**) we compared isolated case where cells don’t communicate by DLL-Notch interaction with interaction case for Ca2+ levels. We observed that secondary oscillation phase rise from interaction dynamics. Similarly, in (**B**) DLL4 concentration elevates for the oscillating high μ interacting cell. Compared to the noninteracting cell with high μ concentration, the interacting cell with the same amount of μ contains significantly higher levels of DLL4.

In the experiments by Yokota et al.^13^, it is observed that DLL4 levels increase as a result of oscillating calcium dynamics and that cells who maintain their oscillatory behavior eventually present a tip phenotype. To demonstrate this phenomenon, temporal simulations are performed. Since tip cell behavior is linked to containing higher DLL4 concentration, Ca2+ has an essential role in gauging the direction such as cell polarization and filopodia stability. During migration, the spatial distribution of calcium is unrelated when phenotype destiny is considered. It is observed that both cells begin to oscillate; during this phase DLL4 concentration in both cells starts to increase. As it can be seen from Fig. 3, Ca2+ oscillations become damped at later times. In some cases, damping may not cease the oscillating completely, but in all cases, it is damped for an interval following primary oscillations. This damping occurs as a result of the lateral inhibition related to HE negative feedback. On the other hand, lateral inhibition decreases the elevated DLL4 levels after oscillations ceased, and hence their contribution is stopped. Following the quiet period, *n* (i.e. the fraction of IP3 receptors which is not closed due to Ca2+) continues to decrease and resurrects oscillations over a certain threshold. DLL4 concentration of the oscillating cell notably increases in the secondary oscillation phase until reaching a steady state notably higher than the other cells. Therefore, the cell having notably higher DLL4 levels is defined as a tip cell.

Here we can observe that DLL4 has small gradual oscillations. This feature takes place since Ca2+ dynamics is much faster than DLL4-Notch interaction.

### Multi-cell Temporal Model

**Figure 4:**
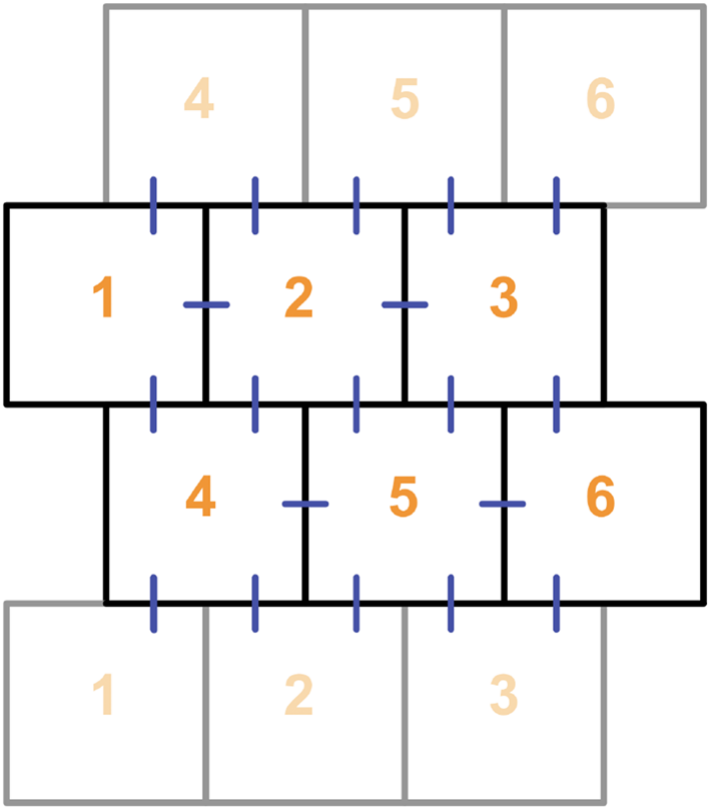
Cell enumeration and placement in the multi-cell scheme. Blue lines indicate the cellular interactions, and the fainter cells are placed for depicting the cylindrical boundary.

**Figure 5:**
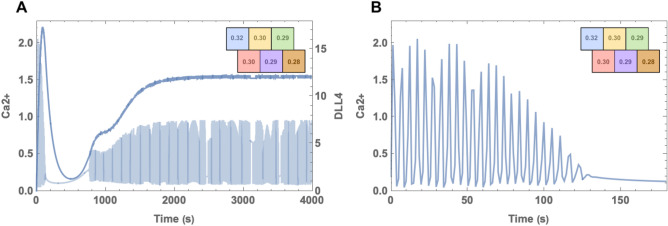
Example simulation for decreasing gradient μ. (**A**) Ca2+ and DLL4 concentration levels for the cell selected as tip phenotype. Oscillating calcium and DLL4 levels are depicted in lighter, and darker tones in the cell color of the cell which can be seen in the top-right. Late calcium oscillations are observed initially in the cell having the highest concentration, $\mu =0.32$. DLL4 concentrations reaching a plateau around t = 1800. (**B**) Calcium dynamics of the selected cells before cessation.

**Figure 6:**
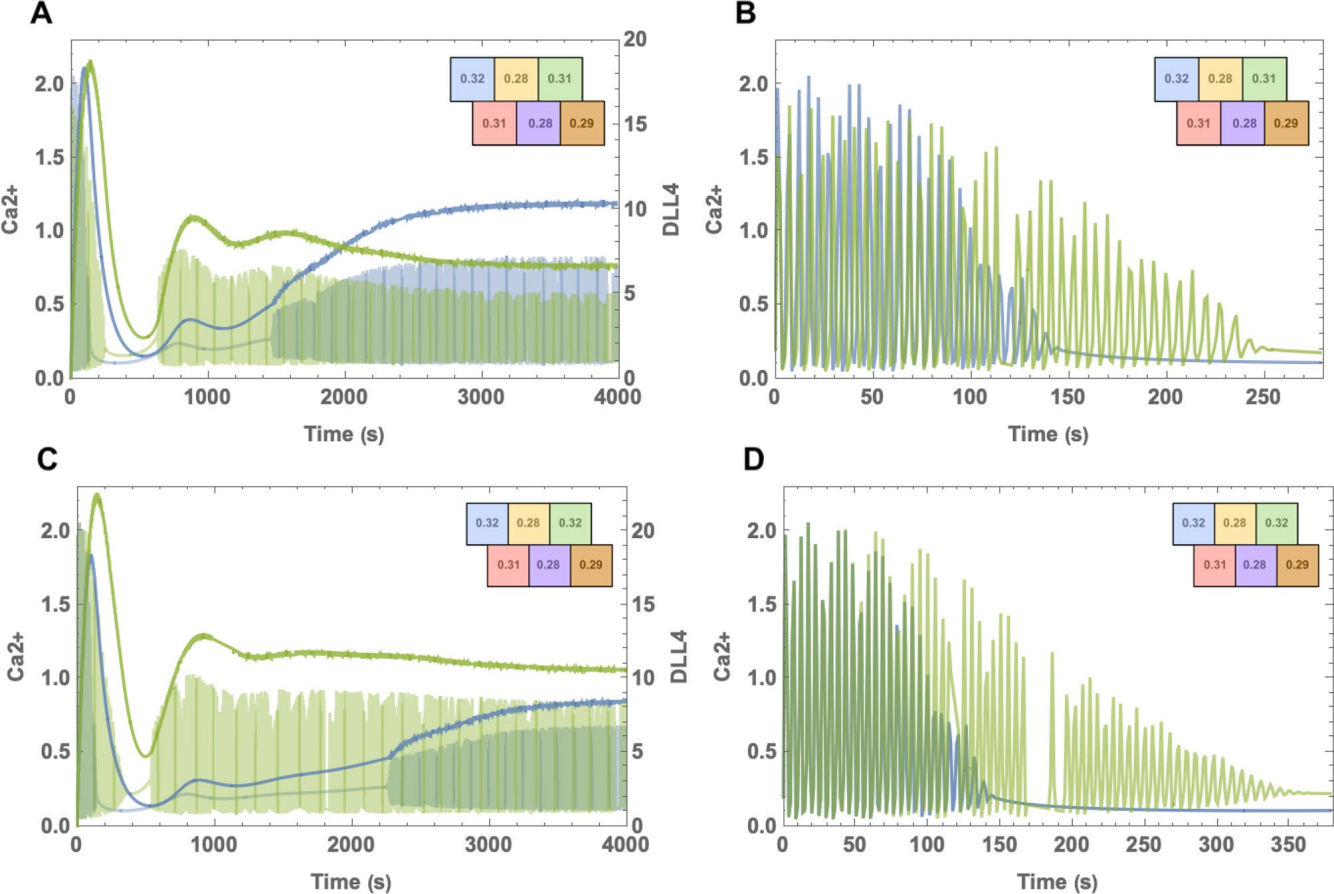
Example scenario in comparison of μ levels. (**A**) Cells containing high μ levels (green and blue) continued to oscillate and selected as tip which are depicted here. Oscillating calcium and DLL4 levels are depicted in lighter, and darker tones in the cell color of the cell which can be seen in the top-right. The cell with the highest concentration is shown to begin oscillating in a later time with lower Ca2+ levels. The late DLL4 concentrations are higher in the blue cell relative to the green. However, it is shown to increase later in accordance with late initiation of Ca2+ oscillation. (**B**) Calcium dynamics of the selected cell before cessation. (**C**) The concentration in the green cell is increased and it is shown to influence the timing for the blue cell’s secondary Ca2+ oscillation phase. DLL4 concentrations for the case where the blue and the green cells contain the same amount of μ. It can be seen that the blue cell has lower DLL4 level for late times. (**D**) With the increased μ levels, calcium oscillation for the green cell lasted longer in comparison with the oscillation in (**B**).

To further elucidate the importance of the interaction dynamics, we simulated various multi-cell scenarios depicting the different levels for VEGF related IP3 intake, μ, and show that calcium oscillating cells remain in the tip character. We aimed to inspect relations between neighbours by a cylindrical vessel comprising 6 cells. Cells are placed as in Fig. 4 where all contacting cells contact the same amount, and interactions are normalized according to the cell number to keep results levelled. We observe that the results of the six cell model agree qualitatively with the results from the two cell model, as can be noticed from the similarity between Figs. 3 and 5. However, due to lateral inhibition from neighbors, we can observe that Ca2+ amplitude in none of the tip cells in multi-cell scenarios were able to reach to the level observed in the two-cell set up. This can be seen from the difference between the two-cell model in Fig. 3 and the multi-cell model in Fig. 6. Although in both of the scenarios, the oscillating tip cell contains the same amount of IP3, calcium reaches lower levels in the secondary oscillatory phase for Fig. 6. Similarly, DLL4 dynamics reached a lower plateau than in the two-cell scenario. This difference occurs due to the lateral inhibition effect arising from the pink neighbour of the tip cell.

For questioning the generalizability of the model, different μ distributions across cells are considered for the following situations. First, the model is evaluated with a decreasing μ gradient towards the cells on the right. Second, for understanding the contribution of cell’s own μ level, we altered the μ level of the cell by increasing only a small amount and compared with the original result. Finally, we analyze a μ distribution allowing the comparison of the cells in the extremities of the vessel. The figures below only include the dynamics of cells selected as tip phenotype; however, figures including Ca2+ and DLL4 dynamics of all cells along with their initial Ca2+ oscillation profiles can be found in supplementary material.

Under a decreasing VEGF gradient in the axial direction, cells select a single cell as having the tip phenotype. When the gradient is applied for higher levels, it is observed that cells reacted in the same manner. It can be observed that in Fig. 5A there is a small change in the increase of the DLL4 level (depicted in darker blue). The change occurs due to the relatively high VEGF intake of the neighbours so that the levels in neighbouring cells are decreased and the tip cell reaches its steady-state earlier. In Fig. 5B, under a decreasing VEGF gradient the multi-cell model ceases oscillating around $t=130$ s before the time calcium oscillation stops around $t= 200$ s.

In Fig. 6 we also examine the contribution of cell μ levels under the same external conditions. In Fig. 6A and B the third (green) cell has $\mu = 0.31$ and in C and D has $\mu = 0.32$. First, it can be seen that the fourth (pink) cell which contains $\mu = 0.31$ as in third (green) cell in Fig. 6A and B, didn’t select the tip cell phenotype under any circumstance. This is due to the higher μ level of the first (blue) cell which is its neighbour. We observed that having higher calcium levels induces higher DLL4 levels in the late period. Another important characteristic of Fig. 6A is that, although μ levels of third (green) cell are lower than the first, Ca2+ oscillation of the third (green) cell started oscillation in an earlier time and from a higher level compared to the first cell. This depicts the influence of neighbour contribution on Ca2+ signalling. Detailed graphics including oscillations of all cells can be seen in supplementary material.

**Figure 7:**
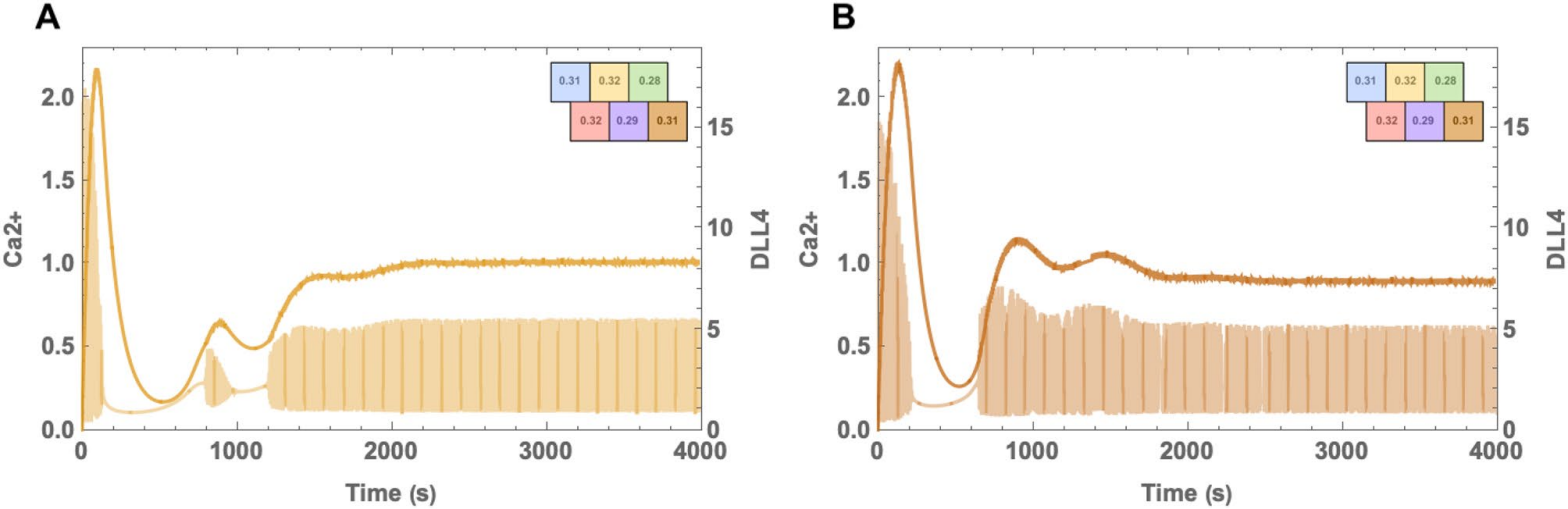
Example scenario where two high μ cells, yellow and pink, are neighbours. (**A**) Ca2+ and DLL4 concentrations for the yellow cell result in selection as tip phenotype. Oscillating calcium and DLL4 levels are depicted in lighter, and darker tones relatively. Graphic colors are chosen regarding the color of the cell which can be seen in the top-right. Calcium oscillates for yellow and orange cells. One of the cells having the highest μ concentration doesn’t regain it’s oscillations. The yellow cell whose neighbours have lower VEGF intake and consequently lower IP3 levels is shown to increase DLL4 levels in the secondary phase. (**B**) Ca2+ and DLL4 concentration levels for the orange cell selected as tip phenotype. Calcium oscillation doesn’t cease for the second time as the tip cell in A. Also, the late DLL4 levels are lower for the orange cell than of yellow cell.

Lastly, to examine the case where the cells with the highest IP3 concentrations are adjacent, we used the distribution shown in Fig. 7. Here cells depicted with yellow and pink colours contain the same highest IP3 levels. The yellow cell wins the competition between the two, and the pink cell didn’t regain Ca2+ oscillations. Although the yellow cell does resume oscillations, as can be observed in Fig. 7A, calcium levels during the oscillation are lower than the levels in the orange cell. It shows the determinant influence of neighbouring dynamics on Ca2+ concentrations. On the other hand, μ levels of the yellow cell reach a higher steady-state than the orange cell similar to our prior observations. In addition, as can been seen in supplementary material, the pink cell oscillates in high levels during the initial oscillation period; however, it’s oscillatory dynamics ceased due to influence by the blue cell. This clearly shows that cell-to-cell communication determines the phenotype decision. This is particularly important since lateral inhibition suggests that neighbouring cells are deterred to posses the tip phenotype altogether.

## Discussion

Calcium plays a crucial role in many steps of cell communication as a second messenger. However, the role of Ca2+ oscillations in EC decision-making has not yet been entirely understood, but its importance as a key molecule is nevertheless clear. Here, by relating two experimentally validated models^25,29^, we conceptualized a calcium-angiogenesis model to describe the relationship between cytosolic Ca2+ and DLL4/Notch dynamics. We employ mathematical and computational models to investigate the significance of certain actors in the angiogenesis signaling pathway. We examine the role of calcium in the context of angiogenesis-related oscillations, which have been shown to play a significant role in early angiogenesis during the phenotype decision making process^13^. Investigation of pathway-specific events can serve as a framework for explaining the observed results.

In the experiments by Yokota et al.^13^, some cells having a longer oscillation duration relative to their neighbors expressed a tip cell phenotype in response to VEGF concentration. For investigating this phenomenon, we simulated cells with the only difference being their cytosolic IP3 concentration, μ (which is related to VEGF concentration). These different IP3 levels determined the overall concentration of DLL4 levels which defines phenotypes. Throughout our simulations, the cell having a higher concentration of μ relative to its neighbors was selected as having a tip cell phenotype. Similarly, we observed that oscillating calcium levels lead to a higher DLL4 concentration and hence, classification as a tip cell. As shown in other studies^14,26^, all cells receiving VEGF signaling altered calcium levels. Cells oscillate initially, but their oscillations cease if a tip cell phenotype is not selected.

In Fig. 2, we observed a directed wave in low μ for the initial oscillation. In contrast to Fig. 2, for high IP3 levels, the initial wave structure is somewhat directionless, though, in later times, oscillations gained directional proceeding wave dynamics. Since Ca2+ is shown to gauge directional features on cell migration, such as filopodia or cytoskeletal remodeling^40^, this observation might be related to complicated vascular configurations in tumor angiogenesis, where ECs are known to have higher levels of VEGF intake relative to healthy angiogenesis. Endothelial cells are known to expand their branches rather than elongating and branching^28^ under high VEGF concentrations. The relation between changing spatial calcium dynamics and obstruction in EC migration might be investigated as a future work.

In Fig. 5, it is observed that under a VEGF gradient, cells containing the highest levels of IP3 due to VEGF intake remain as a tip cell. However, other cells having relatively high IP3 levels fail to gain the phenotype. Another observation is the difference between the influence of neighboring and intrinsic μ levels.

In Fig. 6B and D, it can be observed that tip selection is determined by cellular μ level. However, as observed from the 4th (pink) cell, high levels do not always guarantee that the tip character and neighboring cell IP3 levels become decisive. Another observation is that in the calcium oscillations depicted in Fig. 6A and C , the first cell (blue), is shown to gain its oscillatory behavior in a later time than the third cell (green). This indicates that when resurrecting the oscillations, μ concentrations of the neighbors have higher importance than the cell’s own μ level. Finally, in Fig. 7, we compare oscillations when the two cells having the same concentration are adjacent to each other. Together with cell-to-cell interaction, lateral inhibition enabled only one of the cells to become a stalk cell.

Our model results show how calcium dynamics are a crucial part of phenotype selection. Significantly, calcium is involved in multiple ways in the process of cell migration. From gauging focal adhesion sites with local flickers to retracting the cell with cell-wide transients^9,40,41^, understanding the action of calcium presents opportunities for therapeutic manipulation. Recently, a promising novel cancer therapy called calcium electroporation has been developed which aims to kill cancer cells by increasing cytosolic calcium levels^42^. In addition to eradicating tumor cells, it interferes with the migrational abilities of nearby endothelial cells, which results in anti-vascularization^43^. Models investigating the effects of Ca2+ in cell motility in angiogenesis can be built upon this model.

The widespread usage of the Ca2+ signalling allows it to be used in different areas of the fight against cancer. Although this study doesn’t take into account the role of calcium in cell movement and focuses only on its interaction with DLL4, the fact that calcium signalling plays a role in almost every aspect of cell movement^40,44^ is an element that should be carefully considered. In addition to angiogenesis, cell movement plays a very important role in another cancer related process, metastasis^45,46^. It allows cancer cells to spread to secondary sites after an Epithelial-to-Mesenchymal transition (EMT). The role of Ca2+ signalling in this transformation has been revealed in different types of cancer^47–49^. In addition, studies investigating the relation between DLL’s and metastasis have revealed a possible link^50–52^. Furthermore, Mendonça et al.^53^ showed that inhibition of endothelial specific DLL4/Notch interaction negatively regulates EMT and reduces the number of circulating metastatic cells. Considering these facts, the results in this work might be found interesting in terms of seeking potential therapeutic strategies on EMT prevention.

Nitric oxide (NO), a regulator of microvascular permeability^54^ and apoptosis^55^, is secreted by VEGF-mediated pathways triggered by DLL4 in ECs^56^. For monitoring different mediators of Ca2+, equations representing their kinetics could be added into the model. Along with investigating the permeability of the vessel, such a model would also be useful in commenting on the relation between vessel pruning and EC apoptosis by nitric oxide dependent factors^57,58^. An intriguing extension of this model might also be adding another regulator of calcium homeostasis^59^, mitochondria. It is also known to regulate apoptosis with certain proteins influencing Ca2+ homeostasis^60^ and NO-related factors^61^. These interwoven relationships can be investigated with an additional feedback describing mitochondrial Ca2+ dynamics.

As no mathematical model perfectly represents the entirety of the complex biological interactions at play, our model has a few limitations worth noting. First, the model does not distinguish between the transmembrane and cytosolic proteins; it treats all proteins the same. This enables us to focus only on temporal dynamics between variables. Another limitation is the nature of calcium oscillations: our oscillations have a strong periodic behavior; however, these oscillations have stochastic behaviors in reality. The last limitation is that external influences are implicitly included, such as VEGF concentration and membrane stretching. Since tip cells contain active protrusions, their stretched membranes trigger stretch-activated calcium channels.

The growth of a new vessel begins with phenotype selection at VEGF-activated dormant cells. VEGF activation leads to cell differentiation and concomitant competition between the cells. We focused on the calcium transients observed during these initial processes. By implementing different VEGF intake between the cells as different IP3 concentrations, we showed how VEGF explicitly influences DLL4 levels in cells. With the key elements of the calcium toolbox and cell-to-cell angiogenesis interactions, we made similar observations to experiments. By simulating the model, we showed that calcium oscillations maintained in the cells, which will be determined as tip phenotype, and cell-to-cell interaction between the neighbors influenced calcium levels and phenotype destiny. However, the initial selection triggers migratory changes in the cell. In the future, the role of Ca2+ can be explored more by focusing on the effects of cytoskeletal remodeling during angiogenesis. These new features likely will bring about new feedbacks and pave the way for a more intriguing interplay between phenotype selection and calcium levels.

## Supplementary information

Supplementary Information.

## Data Availability

The authors declare that no competing interests exist.

## References

[1] Hanahan D, Weinberg RA (2000). The hallmarks of cancer. Cell.

[2] Hanahan D, Weinberg RA (2011). Hallmarks of cancer: the next generation. Cell.

[3] Rao N, Lee YF, Ge R (2015). Novel endogenous angiogenesis inhibitors and their therapeutic potential. Acta Pharmacol. Sinica.

[4] Vasudev NS, Reynolds AR (2014). Anti-angiogenic therapy for cancer: current progress, unresolved questions and future directions. Angiogenesis.

[5] Abdalla AM (2018). Current challenges of cancer anti-angiogenic therapy and the promise of nanotherapeutics. Theranostics.

[6] Sosa MS, Bragado P, Aguirre-Ghiso JA (2014). Mechanisms of disseminated cancer cell dormancy: an awakening field. Nat. Rev. Cancer.

[7] Brossa A (2019). Alternative strategies to inhibit tumor vascularization. Int. J. Mol. Sci..

[8] Haibe Y (2020). Resistance mechanisms to anti-angiogenic therapies in cancer. Front. Oncol..

[9] Monteith GR, Prevarskaya N, Roberts-Thomson SJ (2017). The calcium-cancer signalling nexus. Nat. Rev. Cancer.

[10] Iamshanova O, Fiorio Pla A, Prevarskaya N (2017). Molecular mechanisms of tumour invasion: Regulation by calcium signals. J. Physiol..

[11] De Felice D, Alaimo A (2020). Mechanosensitive piezo channels in cancer: focus on altered calcium signaling in cancer cells and in tumor progression. Cancers.

[12] Mo P, Yang S (2018). The store-operated calcium channels in cancer metastasis: from cell migration, invasion to metastatic colonization. Front. Biosci. (Landmark edition).

[13] Yokota Y (2015). Endothelial ca2+ oscillations reflect vegfr signaling-regulated angiogenic capacity in vivo. elife.

[14] Savage AM (2019). tmem33 is essential for vegf-mediated endothelial calcium oscillations and angiogenesis. Nat. Commun..

[15] Page DJ (2019). Positive feedback defines the timing, magnitude, and robustness of angiogenesis. Cell Rep..

[16] Margadant C (2020). Positive and negative feedback mechanisms controlling tip/stalk cell identity during sprouting angiogenesis. Angiogenesis.

[17] Pontes-Quero S (2019). High mitogenic stimulation arrests angiogenesis. Nat. Commun..

[18] Fischer A, Gessler M (2007). Delta-notch-and then? protein interactions and proposed modes of repression by hes and hey bhlh factors. Nucleic Acids Res..

[19] Gerhardt H (2003). Vegf guides angiogenic sprouting utilizing endothelial tip cell filopodia. J. Cell Biol..

[20] Zakirov B (2021). Active perception during angiogenesis: filopodia speed up notch selection of tip cells in silico and in vivo. Philos. Trans. R. Soc. B.

[21] Collier JR, Monk NA, Maini PK, Lewis JH (1996). Pattern formation by lateral inhibition with feedback: a mathematical model of delta-notch intercellular signalling. J. Theor. Biol..

[22] Hellström M (2007). Dll4 signalling through notch1 regulates formation of tip cells during angiogenesis. Nature.

[23] Suchting S (2007). The notch ligand delta-like 4 negatively regulates endothelial tip cell formation and vessel branching. Proc. Natl. Acad. Sci..

[24] Lobov I (2007). Delta-like ligand 4 (dll4) is induced by vegf as a negative regulator of angiogenic sprouting. Proc. Natl. Acad. Sci..

[25] Venkatraman L, Regan ER, Bentley K (2016). Time to decide? dynamical analysis predicts partial tip/stalk patterning states arise during angiogenesis. PLoS One.

[26] Noren DP (2016). Endothelial cells decode vegf-mediated ca2+ signaling patterns to produce distinct functional responses. Sci. Signal..

[27] Bentley K, Chakravartula S (2017). The temporal basis of angiogenesis. Philos. Trans. R. Soc. B Biol. Sci..

[28] Ubezio B (2016). Synchronization of endothelial dll4-notch dynamics switch blood vessels from branching to expansion. Elife.

[29] Atri A, Amundson J, Clapham D, Sneyd J (1993). A single-pool model for intracellular calcium oscillations and waves in the xenopus laevis oocyte. Biophys. J..

[30] Harvey E, Kirk V, Wechselberger M, Sneyd J (2011). Multiple timescales, mixed mode oscillations and canards in models of intracellular calcium dynamics. J. Nonlinear Sci..

[31] Kaouri K, Maini PK, Skourides PA, Christodoulou N, Chapman SJ (2019). A simple mechanochemical model for calcium signalling in embryonic epithelial cells. J. Math. Biol..

[32] Kobayashi Y, Sawabu Y, Kitahata H, Denda M, Nagayama M (2016). Mathematical model for calcium-assisted epidermal homeostasis. J. Theor. Biol..

[33] Kobayashi Y (2014). Mathematical modeling of calcium waves induced by mechanical stimulation in keratinocytes. PLoS One.

[34] Olson SD, Suarez SS, Fauci LJ (2010). A model of catsper channel mediated calcium dynamics in mammalian spermatozoa. Bull. Math. Biol..

[35] Shi X, Zheng Y, Liu Z, Yang W (2008). A model of calcium signaling and degranulation dynamics induced by laser irradiation in mast cells. Chin. Sci. Bull..

[36] Wilkins M, Sneyd J (1998). Intercellular spiral waves of calcium. J. Theor. Biol..

[37] Stepanova D, Byrne HM, Maini PK, Alarcón T (2021). A multiscale model of complex endothelial cell dynamics in early angiogenesis. PLOS Comput. Biol..

[38] Logg A, Wells GN, Hake J (2012). DOLFIN: a C++/Python Finite Element Library, chap. 10.

[39] Alnæs, M. S. *et al.* The fenics project version 1.5. *Archive Numer. Softw.* **3** (2015).

[40] Wei C (2009). Calcium flickers steer cell migration. Nature.

[41] Marchi S, Giorgi C, Galluzzi L, Pinton P (2020). Ca2+ fluxes and cancer. Mol. cell.

[42] Frandsen SK (2012). Direct therapeutic applications of calcium electroporation to effectively induce tumor necrosis. Cancer Res..

[43] Staresinic B (2018). Effect of calcium electroporation on tumour vasculature. Sci. Rep..

[44] Lee J, Ishihara A, Oxford G, Johnson B, Jacobson K (1999). Regulation of cell movement is mediated by stretch-activated calcium channels. Nature.

[45] Welch DR, Hurst DR (2019). Defining the hallmarks of metastasis. Cancer research.

[46] Stuelten CH, Parent CA, Montell DJ (2018). Cell motility in cancer invasion and metastasis: insights from simple model organisms. Nat. Rev. Cancer.

[47] Davis FM (2014). Induction of epithelial-mesenchymal transition (emt) in breast cancer cells is calcium signal dependent. Oncogene.

[48] Norgard, R. J. *et al.* Calcium signaling induces a partial emt. *EMBO Rep.* e51872 (2021). 10.15252/embr.202051872 PMC8419705 34324787

[49] Qi L (2016). Fgf4 induces epithelial-mesenchymal transition by inducing store-operated calcium entry in lung adenocarcinoma. Oncotarget.

[50] Wieland E (2017). Endothelial notch1 activity facilitates metastasis. Cancer Cell.

[51] Huang QB (2014). Endothelial delta-like 4 (dll4) promotes renal cell carcinoma hematogenous metastasis. Oncotarget.

[52] Kuramoto T (2012). Dll4-fc, an inhibitor of dll4-notch signaling, suppresses liver metastasis of small cell lung cancer cells through the downregulation of the nf-κb activity. Mol. Cancer Therap..

[53] Mendonça L (2019). Metastasis is impaired by endothelial-specific dll4 loss-of-function through inhibition of epithelial-to-mesenchymal transition and reduction of cancer stem cells and circulating tumor cells. Clin. Exp. Metast..

[54] Kubes P, Granger DN (1992). Nitric oxide modulates microvascular permeability. Am. J. Physiol. Heart Circ. Physiol..

[55] Kim PK, Zamora R, Petrosko P, Billiar TR (2001). The regulatory role of nitric oxide in apoptosis. Int. Immunopharmacol..

[56] Miloudi K (2019). Notch1 signaling induces pathological vascular permeability in diabetic retinopathy. Proc. Natl. Acad. Sci..

[57] Snyder CM, Shroff EH, Liu J, Chandel NS (2009). Nitric oxide induces cell death by regulating anti-apoptotic bcl-2 family members. PloS one.

[58] Watson E, Whitehead L, Adams R, Dewson G, Coultas L (2016). Endothelial cell survival during angiogenesis requires the pro-survival protein mcl1. Cell Death Differ..

[59] Dupont G, Falcke M, Kirk V, Sneyd J (2016). Models of Calcium Signalling.

[60] Bock FJ, Tait SW (2020). Mitochondria as multifaceted regulators of cell death. Nat. Rev. Mol. Cell Biol..

[61] Boyd, C. S. & Cadenas, E. Nitric oxide and cell signaling pathways in mitochondrial-dependent apoptosis (2002). 10.1515/BC.2002.045 12033432

