# Supplementary material for: The role of calcium oscillations in the phenotype selection in endothelial cells
Source: Sci Rep. 2021 Dec 10;11:23781. doi: 10.1038/s41598-021-02720-2 (PMC8664853; doi:10.1038/s41598-021-02720-2)
Supplement: Supplementary file 1 — Supplementary Information. [file 41598_2021_2720_MOESM1_ESM.pdf]

Supplementary Information for The Role of Calcium Oscillations in the Phenotype Selection in Endothelial Cells

Birses Debir[1,*], **Cameron Meaney**[2], **Mohammad Kohandel**[2], **and M. Burcin Unlu**[1,3]

[1]Bogazici University, Department of Physics, Istanbul, 34342, Turkey
[2]University of Waterloo, Department of Applied Mathematics, Waterloo, ON, Canada
[3]Hokkaido University, Global Station for Quantum Medical Science and Engineering, Global Institution for Collaborative Research and Education (GI-CoRE), Sapporo, 060-8648, Japan
[*]birses.debir@gmail.com

Calcium and DLL4 Dynamics of All Cells

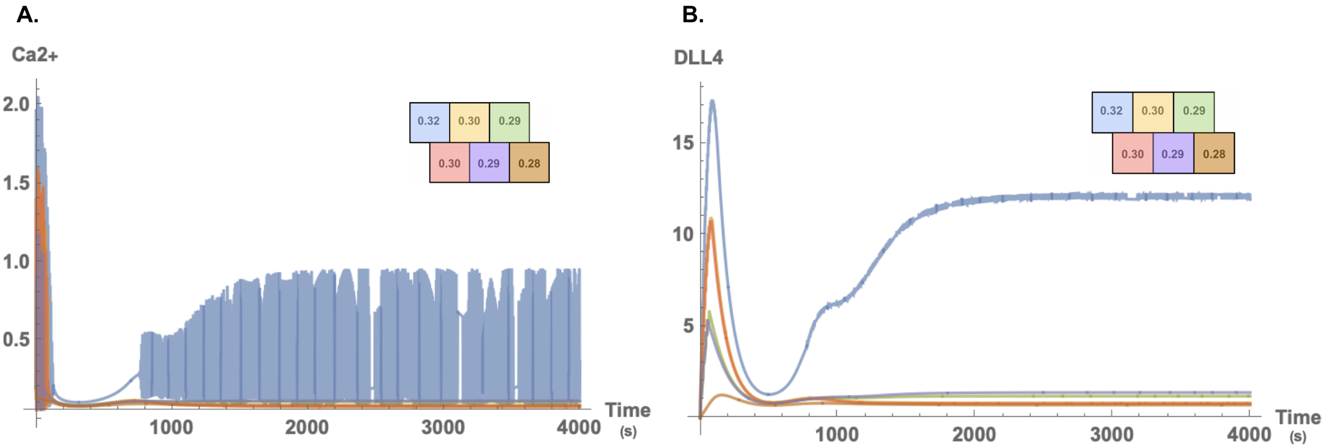

Figure 1. Example simulation for decreasing gradient μ. **(A)** Late calcium oscillations are observed initially in the cell having the highest concentration, $\mu = 0.32$. **(B)** DLL4 concentrations reaching a plateau around t=1800.

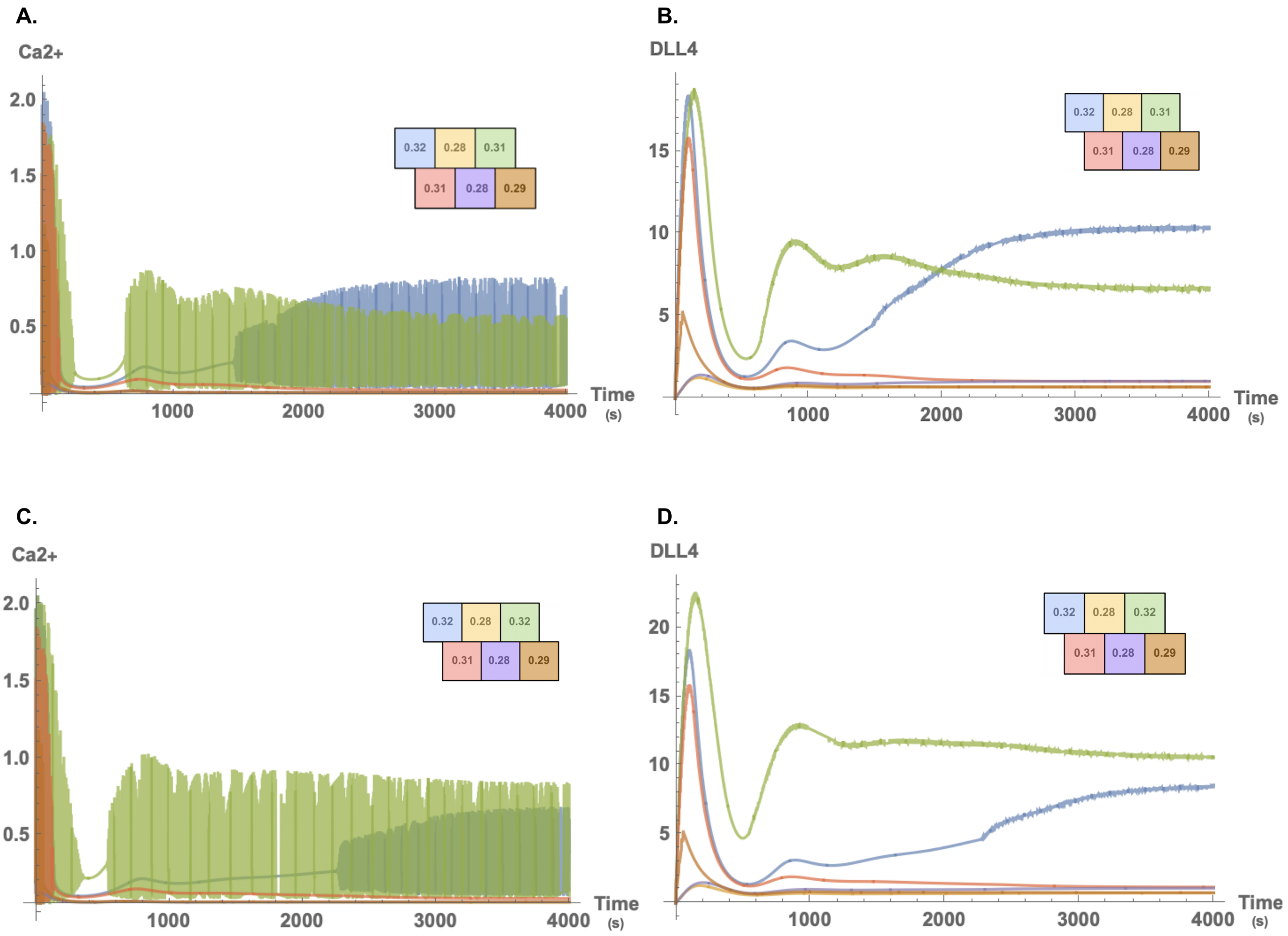

Figure 2. Example scenario in comparison of μ levels. **(A)** Cells containing high μ levels (green and blue) continued to oscillate. The cell with the highest concentration is shown to begin oscillating in a later time with lower Ca2+ levels. Although having the same amount of μ, the 4th cell (pink) doesn't posses continual oscillations as in the green cell. **(B)** The late DLL4 concentration are higher in the blue cell relative to the green. However it is shown to increase later in accordance with late initiation of Ca2+ oscillation. **(C)** The concentration in the green cell is increased and it is shown to influence the timing for blue cells secondary Ca2+ oscillation phase. **(D)** DLL4 concentrations for the case where the blue and the green cells contains same amount of μ. It can be seen that the blue cell has lower DLL4 level for late times.

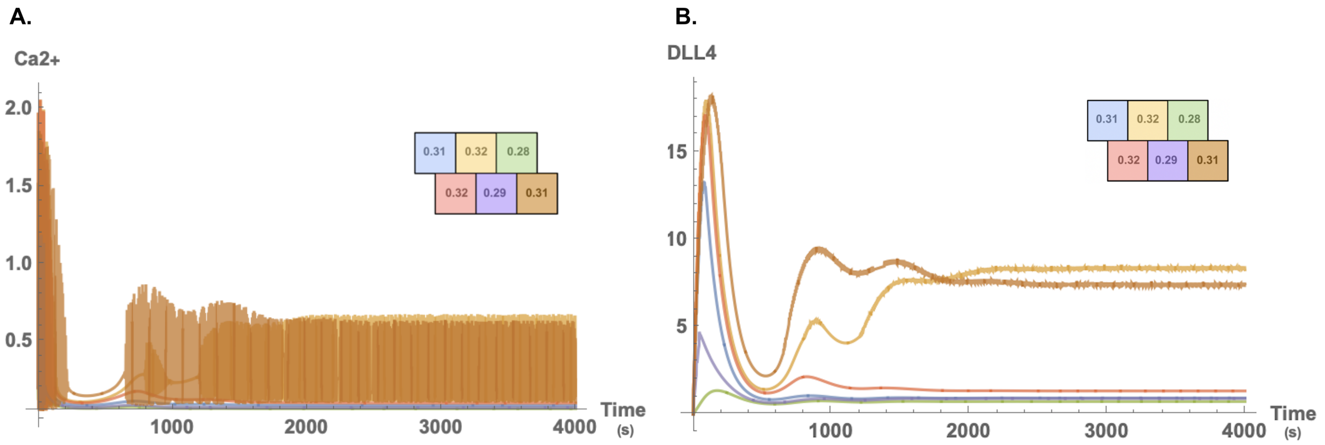

Figure 3. Example scenario where two high μ cell yellow and pink are neighbours. **(A)** Calcium oscillates for yellow and orange cells. One of the cells having the highest μ concentration doesn't regain it's oscillations. **(B)** It can be seen from DLL4 concentrations is that the pink cell (the cell with stalk phenotype despite the high VEGF intake) increased to similar DLL4 levels with the yellow cell, yet unable to re-increase the concentration. However, the yellow cell whose neighbours have lower VEGF intake and consequently lower IP3 levels is shown to increase DLL4 levels in the secondary phase. Also, the late DLL4 levels are higher for the yellow cell than of orange cell.

Pre-cessation Calcium Dynamics of All Cells

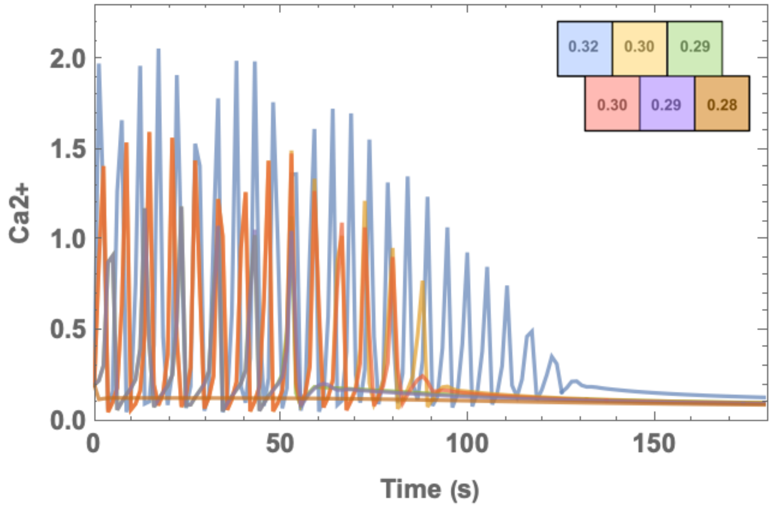

Figure 4. Enlarged Ca2+ dynamics before cessation of above cells.

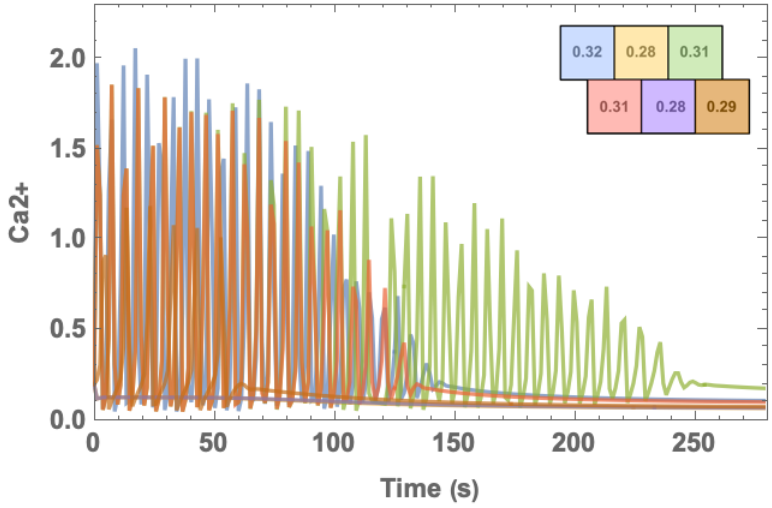

Figure 5. Enlarged Ca2+ dynamics before cessation of above cells.

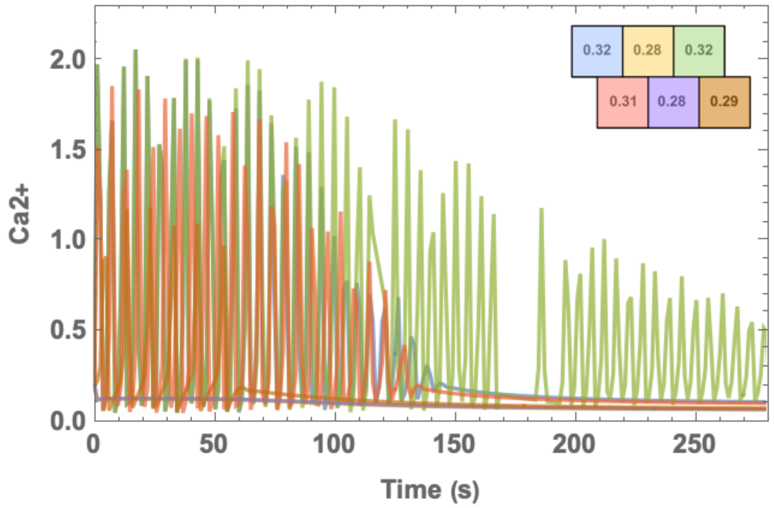

Figure 6. Enlarged Ca2+ dynamics before cessation of above cells.

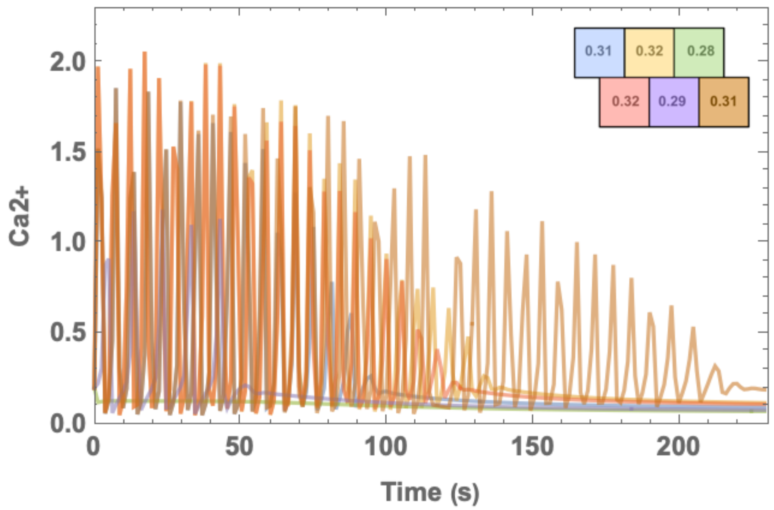

Figure 7. Enlarged Ca2+ dynamics before cessation of above cells.
